# Distinct eLPB^ChAT^ projections for methamphetamine withdrawal anxiety and primed reinstatement of conditioned place preference

**DOI:** 10.7150/thno.95383

**Published:** 2024-04-29

**Authors:** Wenwen Chen, Hao Guo, Ning Zhou, Yuning Mai, Tao Hu, Xing Xu, Teng He, Jun Wen, Shan Qin, Chengyong Liu, Wenzhong Wu, Hee Young Kim, Yu Fan, Feifei Ge, Xiaowei Guan

**Affiliations:** 1 Department of Human Anatomy and Histoembryology, Nanjing University of Chinese Medicine, Nanjing, China.; 2 Department of Acupuncture-Moxibustion and Rehabilitation, Jiangsu Province Hospital of Chinese Medicine, Affiliated Hospital of Nanjing University of Chinese Medicine, Nanjing, China.; 3 Department of Physiology, Yonsei University College of Medicine, Seoul, South Korea.

**Keywords:** eLPB^ChAT^-lCeA^PKCδ^, eLPB^ChAT^-ovBNST^PKCδ^, methamphetamine, anxiety, conditioned place preference

## Abstract

Methamphetamine (METH) withdrawal anxiety symptom and relapse have been significant challenges for clinical practice, however, the underlying neuronal basis remains unclear. Our recent research has identified a specific subpopulation of choline acetyltransferase (ChAT^+^) neurons localized in the external lateral portion of parabrachial nucleus (eLPB^ChAT^), which modulates METH primed-reinstatement of conditioned place preference (CPP). Here, the anatomical structures and functional roles of eLPB^ChAT^ projections in METH withdrawal anxiety and primed reinstatement were further explored.

**Methods:** In the present study, a multifaceted approach was employed to dissect the LPB^ChAT+^ projections in male mice, including anterograde and retrograde tracing, acetylcholine (Ach) indicator combined with fiber photometry recording, photogenetic and chemogenetic regulation, as well as electrophysiological recording. METH withdrawal anxiety-like behaviors and METH-primed reinstatement of conditioned place preference (CPP) were assessed in male mice.

**Results:** We identified that eLPB^ChAT^ send projections to PKCδ-positive (PKCδ^+^) neurons in lateral portion of central nucleus of amygdala (lCeA^PKCδ^) and oval portion of bed nucleus of the stria terminalis (ovBNST^PKCδ^), forming eLPB^ChAT^-lCeA^PKCδ^ and eLPB^ChAT^-ovBNST^PKCδ^ pathways. At least in part, the eLPB^ChAT^ neurons positively innervate lCeA^PKCδ^ neurons and ovBNST^PKCδ^ neurons through regulating synaptic elements of presynaptic Ach release and postsynaptic nicotinic acetylcholine receptors (nAChRs). METH withdrawal anxiety and METH-primed reinstatement of CPP respectively recruit eLPB^ChAT^-lCeA^PKCδ^ pathway and eLPB^ChAT^-ovBNST^PKCδ^ pathway in male mice.

**Conclusion:** Our findings put new insights into the complex neural networks, especially focusing on the eLPB^ChAT^ projections. The eLPB^ChAT^ is a critical node in the neural networks governing METH withdrawal anxiety and primed-reinstatement of CPP through its projections to the lCeA^PKCδ^ and ovBNST^PKCδ^, respectively.

## Introduction

Methamphetamine (METH) is a highly addictive and widely abused psychostimulant drug. Among individuals with METH use disorders (MUD), it has been observed that 34.3% develop withdrawal anxiety symptoms 1, 2, and up to 90% are estimated to relapse 3, posing a significant challenge for clinical practice.

The parabrachial nucleus (PBN), being located within the pons, functions as an integrator of sensory input from the surrounding environment and subsequently relays this information to forebrain structures. The majority of PBN neurons are excitatory features 4, 5, being positively correlated with behaviors indicative of pain 6-8, itch 9, 10, fear 11, aversive 12, and emotion 10, 13. Our recent research has identified a specific subpopulation of choline acetyltransferase (ChAT^+^) neurons located in the PBN, specifically within the external lateral portion of PBN (eLPB^ChAT^) 14. These neurons play a crucial role in modulating METH-primed reinstatement of METH conditioned place preference (CPP) in male mice. However, the precise structures and functions of eLPB^ChAT^ projections have not yet to be explored.

The LPB predominately send projections to the thalamus and limbic nuclei, such as the central nucleus of the amygdala (CeA) 6, 8, 13 and the bed nucleus of the stria terminalis (BNST) 6, 15, 16, both of which are pivotal in anxiety 13, 17 and addiction 18-20. The activation of projections from the LPB to the CeA or BNST elicits aversive responses 6. Given its potential as a target for anxiolytic agents, the CeA is implicated in the emotional component of the behavioral reaction to alcohol, particularly anxiety 21, 22. Activation of LPB-CeA pathway alone was sufficient to induced anxiety-like behavior in mice 13. It is noteworthy that social choice-induced abstinence has been found to prevent the incubation of METH craving, which was associated with the activation of protein kinase C delta-positive neurons in the CeA (CeA^PKCδ^) 19. Furthermore, withdrawal from METH has been shown to increase the activity of BNST neurons 23, and BNST^PKCδ^ neurons are also activated by aversive conditions to promote anxiety-like behavior 17. Based on the mapping results obtained from fMOST 14, our previous findings have identified two distinct groups of eLPB^ChAT^ efferent neurons projecting to the CeA and BNST. Taken together, we aimed to gain a more comprehensive understanding of LPB^ChAT+^ projections and their potential contribution to METH-related behaviors.

In the present study, a multifaceted approach was employed to dissect the LPB^ChAT+^ projections in mice, including anterograde and retrograde tracing, acetylcholine (Ach) indicator combined with fiber photometry recording, as well as electrophysiological recording. We first identified the structural connectivity and physiological innervation of eLPB^ChAT^-CeA pathway and eLPB^ChAT^-BNST pathway in naïve male mice. Then, we explored the role of eLPB^ChAT^-CeA pathway and eLPB^ChAT^-BNST pathway in the anxiety-like behaviors during METH withdrawal period and METH-primed reinstatement of CPP in male mice. Our findings put new insights into the complex neural networks, especially focusing on the eLPB^ChAT^ projections, that contribute to the METH withdrawal anxiety and primed reinstatement of CPP.

## Materials and methods

### Animals

Male C57BL/6 wild type (WT) and *ChAT-Cre* male mice weighing 22-25 g (9 weeks old) were used. All animals were housed at constant humidity (40~60%) and temperature (24 ± 2ºC) with a 12-hour light/dark cycle (lights on at 8 a.m.) and allowed free access to food and water. All male mice were handled for three days before onset of experiments. The naive male mice utilized in this experiment were not subjected to any drug treatment or behavioral training, and had an initial weight range of 22-25g (9 weeks old). All procedures were carried out in accordance with the National Institutes of Health Guide for the Care and Use of Laboratory Animals and approved by the Institutional Animal Care and Use Committee (IACUC) at Nanjing University of Chinese Medicine, China.

### Immunofluorescence

The male mice were deeply anesthetized with 10% chloral hydrate (0.2 ml, i.p.) and sequentially perfused with 0.9% saline and 4% paraformaldehyde (PFA). The brains were removed and post-fixed in 4% PFA at 4 °C overnight. After dehydration of the brains with 30% (w/v) sucrose, coronal brain sections (30 μm) were cut on a cryostat (Leica, Germany) and used for immunofluorescence. The sections were incubated in 0.3% (v/v) Triton X-100 for 0.5 h, blocked with 5% donkey serum for 1.5 h at room temperature, and incubated overnight at 4°C with the following primary antibodies: goat anti-ChAT (1:200, RRID: AB_2079751, Millipore, USA), mouse anti-NeuN (1:800, RRID: AB_2298772, Millipore, USA), guinea pig anti-c-Fos (1:3000, RRID: AB_2905595, Synaptic System, USA), rabbit anti-c-Fos (1:2000, RRID: AB_2247211, Cell Signaling Technology, USA), mouse anti-c-Fos (1:1500, RRID: AB_2747772, Abcam, USA) and rabbit anti-PKCδ (1:1000, RRID: None, HuaBio, China), followed by the corresponding fluorophore-conjugated secondary antibodies for 1.5 h at room temperature. The following secondary antibodies were used here: Alexa Fluor 488-labeled donkey anti-rabbit secondary antibody (1:500, RRID: AB_2762833, Invitrogen, USA), Alexa Fluor 488-labeled donkey anti-mouse secondary antibody (1:500, RRID: AB_141607, Invitrogen, USA), Alexa Fluor 555-labeled goat anti-guinea pig secondary antibody (1:500, RRID: None, Abcam, USA), Alexa Fluor 555-labeled donkey anti-rabbit secondary antibody (1:500, RRID: AB_2762834, Invitrogen, USA), Alexa Fluor 555-labeled donkey anti-mouse secondary antibody (1:500, RRID: AB_2762848, Invitrogen, USA), Alexa Fluor 555-labeled donkey anti-goat secondary antibody (1:500, RRID: AB_2762839, Invitrogen, USA), DyLight™ 680-labeled goat anti-guinea pig secondary antibody (1:500, RRID: AB_2556678, Invitrogen, USA), Alexa Fluor 680-labeled donkey anti-rabbit secondary antibody (1:500, RRID: AB_2762836, Invitrogen, USA), Alexa Fluor 680-labeled donkey anti-mouse secondary antibody (1:500, RRID: AB_2762831, Invitrogen, USA), Alexa Fluor 680-labeled donkey anti-goat secondary antibody (1:500, RRID: AB_2762841, Invitrogen, USA). In detail of the staining for c-Fos and PKCδ in the lCeA or ovBNST, brain slices were attached to slides and placed in an oven at 60°C for 30 min, and then immersed in 4% PFA solution for 15 min in a refrigerator at 4°C for fixation. Then graded concentration alcohol dehydration (50%-70%-100%-100%) was performed sequentially for 5 min each time. Next, antigen repair was performed by immersing the slides with brain slices attached in 10 mM PH=6.0 trisodium citrate solution and heating in a water bath at 82°C for 30 min. Normal staining steps were then followed. Fluorescence signals were captured by TCS SP8 confocal microscope (Leica, Germany), and STELLARIS 8 DIVE confocal microscope (Leica, Germany).

### Tracing virus injection

All viruses in the present study were packaged by BrainVTA (China). The male mice were fixed in a stereotactic frame (RWD, China) under 2% isoflurane anesthesia. A heating pad was used to maintain the body temperature of the mice at 37 °C. Unless otherwise noted, a volume of 100 nl virus was injected per side. The injections were given over 5 min at a rate of 20 nl/min by an infusion pump (Drummond, USA) and left in place for 10 min. The stereotaxic coordinates for the eLPB are used as following: AP, - 5.07/5.19/5.33 mm, ML, ± 1.45 mm and DV, - 3.30 mm. The stereotaxic coordinates of the lCeA are used as following: AP, - 1.31 mm; ML, ± 2.90 mm; DV, - 4.50 mm. The stereotaxic coordinates for the ovBNST are used as following: AP, + 0.13 mm, ML, ± 1.1mm, and DV, - 4.05 mm. For anterograde tracing, the *rAAV2/9-EF1α-DIO-EGFP* (PT-0795, 2.04E+12 vg/ml, BrainVTA, China) virus was injected into the unilateral eLPB of ChAT-Cre mice. For retrograde tracing, the CTB-555 (CTB-02, 1 μg/μl, BrainVTA, China) was injected into the unilateral lCeA and ovBNST of WT mice. After 1-week (retrograde tracing) and 3/4-week (anterograde tracing) transfection, male mice were perfused with 0.9% saline, followed by 4% PFA and then images of the CTB-555 or EGFP signals were visualized to assess the virus-injected positions. Animals with missed injections were excluded from the study.

### Fiber photometry

On WT male mice, the *rAAV2/9-hSyn-Ach3.0* (PT-1335, 5.68E+12 vg/mL, BrainVTA, China) virus was unilaterally injected into the lCeA (AP, - 1.31 mm; ML, - 2.90 mm; DV, - 4.50 mm) or ovBNST (AP + 0.13 mm, ML - 1.1mm, DV - 4.05 mm), while the* rAAV2/9-ChAT-hM3D(Gq)-mCherry* (PT-2213, 5.54E+12 vg/ml, BrainVTA, China) or* rAAV2/9-ChAT-hM4D(Gi)-mCherry* (PT-3108, 6.28E+12 vg/ml, BrainVTA, China) virus was bilaterally injected into the eLPB (AP, - 5.19 mm, ML, ± 1.45 mm and DV, - 3.30 mm). An optical fiber (200 μm outer diameter, 0.37 numerical aperture, Thinkerbiotech, China) was placed 100 μm above the viral injection site. After 3 weeks, the calcium-dependent fluorescence signals of Ach 3.0 sensor in the lCeA or ovBNST were recorded at homecage. The signals were obtained by stimulating cells that transfected Ach3.0 sensor using a 470 nm LED (35-40 μW at fiber tip), while calcium-independent signals were obtained by stimulating these cells with a 405 nm LED (15-20 μW at fiber tip). The LED lights of 470 nm and 405 nm were alternated at 66 fps and light emission was recorded using sCMOS camera containing the entire fiber bundle (2 m in length, NA = 0.37, 200 μm core, Thinkerbiotech, China). The analog voltage signals fluorescent was filtered at 30 Hz and digitalized at 100 Hz. The Ach3.0 signals were recorded and analyzed by ThinkerTech TrippleColor MultiChannel fiber photometry Acquisition Software and ThinkerTech TrippleColor MultiChannel fiber photometry Analysis Package (Thinkerbiotech, China), respectively. The raw heatmap data were normalized by Z-Score normalization. The formula for Z-Score is (D - μ)/σ, where D is the raw fiber photometry signal data, μ is the mean value of raw data and σ is the standard deviation of raw data. The baseline fluorescence signal which was recorded for 5 min with 1 min record and 4 min interval (1 session) prior to clozapine-N-oxide (CNO, Selleck, USA) treatment. The real-time fluorescence signal which was recorded for 60 min with a 1-min recording and 4-min interval (11 session). The raw heatmap data from one mouse was merged as a statistical point and normalized using area under the curve (AUC) normalization. The AUC represents the integral under the recording duration relative to the corresponding baseline at each trial. For the immunofluorescence experiment, another set of same mice models was utilized, the brain tissue was collected at 35 min after CNO injection.

### Electrophysiology

The* rAAV2/9-ChAT-CRE-P2A-EGFP-WPRE-hGH* polyA (PT-0652, 5.30E+12 vg/mL, BrainVTA, China) and *rAAV2/9-Ef1α-DIO-hChR2-mCherry-WPRE-hGH pA* (PT-0002, 5.05E+12 vg/mL, BrainVTA, China) virus was bilaterally injected into the eLPB of WT mice. The mice (n = 6 mice) were deeply anesthetized with isoflurane (RWD, China) and perfused with ice-cold cutting solution. Slice preparation was performed as previously described 2. Slices containing the lCeA or ovBNST were cut at a 200 μm thickness using a vibratome in 4°C cutting solution. The slices were transferred to 37°C cutting solution for 9 min, then transferred to a holding solution to allow for recovery at room temperature for 1 h before recordings. Throughout the electrophysiological recordings, the slices were continuously perfused with oxygenated artificial CSF (aCSF) maintained at 30°C using a solution heater (TC-324C, Warner Instruments, USA).

The spontaneous excitatory postsynaptic currents (sEPSC) were recorded in voltage-clamp mode at a holding potential of -70 mV. The sEPSC baseline was recorded 5 min after breaking in. An LED fiber above the recording sites emits blue (473 nm) light to activate the terminals of the eLPB^ChAT^ neurons within the lCeA or ovBNST. Mecamylamine (MEC, 5 μM, nicotinic actelycholine receptors antagonist) was used to non-specifically inhibit nicotinic acetylcholine receptors (nAChRs) 24.

All signals were filtered at 4 kHz, amplified at 5× using a MultiClamp 700B amplifier (Molecular Devices, USA) and digitized at 10 kHz with a Digidata 1550B analog-to-digital converter (Molecular Devices, USA). All data were analyzed with Clampfit 10.7 software (Molecular Devices, USA).

### Elevated plus maze (EPM)

In the EPM experiment, male mice were assigned to receive a daily intraperitoneal injection of METH (METH group, 3 mg/kg) or saline (SAL group, 0.2 ml) for 7 consecutive days, followed by a 14-day abstinence (prolonged abstinence) period. On day 22, the EPM was performed to examine anxiety-like behaviors in male mice. The EPM apparatus (TopScan, USA) is composed of 4 elevated arms (52 cm above the floor) arranged in a cross pattern with two 1-cm walled arms (open arms) and two 40-cm walled arms (closed arms). Each male mouse was placed in the center portion of the EPM with its head directed towards an open arm, and allowed to freely explore the maze for 5 min. The time spent in and the entries into the open arms, as well as the total distance traveled within the apparatus were recorded and analyzed using TopScan H system (TopScan, USA).

For chemical genetics stimulations of the eLPB^ChAT^ terminals within the lCeA, the *rAAV2/9-ChAT-hM4D(Gi)-mCherry* (PT-3108, 6.28E+12 vg/ml, BrainVTA, China) virus was bilaterally injected into the eLPB (AP ± 5.19 mm, ML ± 1.45 mm, DV - 3.30 mm), and stainless-steel guide cannulas (O.D. 0.41 mm, C.C 4.0 mm, Cat 62004, RWD, China) were lowed into the lCeA (AP - 1.31 mm, ML ± 2.90 mm, DV - 4.50 mm). The guide cannulas were secured in place using glass ionomer cements. Dummy cannulas (62102, RWD, China, with lengths matching the guide cannulas) were placed inside the guide cannulas to prevent occlusion. Incisions were fixed and covered with glass ionomer cement.

### Conditioned place preference (CPP)

The CPP test was performed in the TopScan3D CPP apparatus (CleverSys, USA), consisting of two distinct chambers (15 × 15 × 23 cm each) with a removable guillotine door. The CPP procedure involved a conditioning test (Pre-test, Day -1), conditioning (CPP training, Day 0-6), post-conditioning test (Test, Day 7), extinction training, and a saline or METH challenge-primed reinstatement test. Baseline preference (pre-test) was determined by allowing male mice to freely explore both chambers of the CPP apparatus for 15 min. Conditioning was conducted in a non-drug-paired chamber paired with a saline (0.2 ml, i.p.) injection in the morning and in a drug-paired chamber paired with a METH (3 mg/kg, i.p.) injection in the afternoon for 7 consecutive days. Following each injection, the male mice were confined to one chamber (non-drug-paired chamber or drug-paired chamber) for 45 min. During the test and extinction phases, male mice had unrestricted access to both chambers without any drug treatment. For the METH-primed reinstatement test, male mice received a subthreshold does of METH (0.5 mg/kg, i.p.) and were then allowed to freely explore both chambers for 15 min. The CPP score represents the time spent in drug-paired chamber minus that in the non-drug-paired chamber, while the ΔCPP score is calculated as the priming CPP score minus extinction CPP score.

In the chemical genetics stimulations experiment, the* rAAV2/9-ChAT-hM3D(Gq)-mCherry* (PT-2213, 5.54E+12 vg/ml, BrainVTA, China) or *rAAV2/9-ChAT-hM4D(Gi)-mCherry* (PT-3108, 6.28E+12 vg/ml, BrainVTA, China) virus was bilaterally injected into the eLPB (AP ± 5.19 mm, ML ± 1.45 mm, DV - 3.30 mm), and the cannulas were lowed into the lCeA (AP - 1.31 mm, ML ± 2.90 mm, DV - 3.0 mm) (cannula: O.D. 0.41 mm, C 4.0 mm, Cat 62004, RWD, China) or ovBNST (AP + 0.13 mm, ML ± 2.13 mm, DV - 2.62 mm, 15°tilt) (cannula: O.D. 0.41 mm, C 3.6 mm, Cat 62004, RWD, China).

### Designer receptors exclusively activated by designer drugs (DREADDs)

Male mice were anesthetized with 2% isoflurane in oxygen and were fixed in a stereotactic frame (RWD, China). A heating pad was used to maintain the core body temperature of the animals at 37°C. 100 nl *rAAV2/9-ChAT-hM3D(Gq)-mCherry* (PT-2213, 5.54E+12 vg/ml, BrainVTA, China) or *rAAV2/9-ChAT-hM4D(Gi)-mCherry* (PT-3108, 6.28E+12 vg/ml, BrainVTA, China) virus was bilaterally injected into the eLPB (AP ± 5.19 mm, ML ± 1.45 mm, DV - 3.30 mm) at a rate of 1 nl/sec. After surgery, mice were allowed to recover in their homecage for one week. 10 min before the behavioral assays, CNO or saline was locally infused through the cannula at a flow rate of 0.1 µl/min. The infusion cannulas (#62204, RWD, China) were connected via polyethylene tubing (#62329, RWD, China) to 10-μl microsyringes (GAOGE, China) mounted on a microinfusion pump (RWDR462, China). For the diffusion of the drug, the infusion cannulas were kept in place for 5 min before being replaced with dummy cannulas. Male mice were injected with saline (vehicle, 0.2 ml) or CNO (2 mg/kg) 10 min before each behavioral test.

### Statistical analysis

Statistical analysis was carried out using GraphPad Prism 8.0.2 software. All data are presented as the Mean ± SD. The data were analyzed by unpaired *t*-tests or two-way analysis of variance (ANOVA) with Sidak's multiple comparisons which appropriate. All statistical significance was set as *p* < 0.05.

## Results

### Anatomical dissection and functional investigation of the eLPB^ChAT^-lCeA^PKCδ^ and eLPB^ChAT^-ovBNST^PKCδ^ pathways

**Figure 1:**
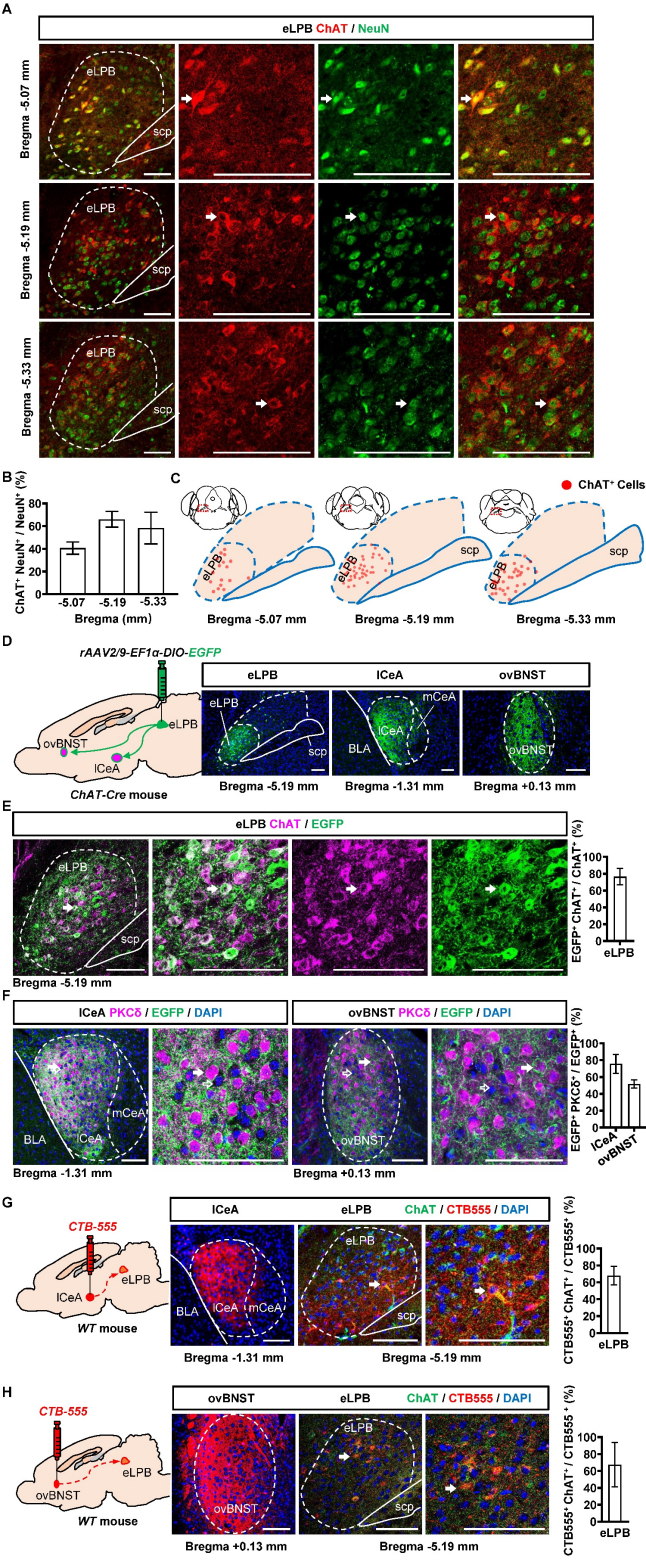
** Anatomical structure of the eLPB^ChAT^-lCeA^PKCδ^ and eLPB^ChAT^-ovBNST^PKCδ^ pathways in naïve male mice. A, B** Immunohistochemistry for ChAT/NeuN in the eLPB of WT mice. Scale bar, 100 μm. **C** Representative distribution images of ChAT neural ensemble in the eLPB. **D** Schematic diagram and representative images of the *rAAV2/9-DIO-EGFP* injection in the eLPB, lCeA and ovBNST in ChAT-Cre mouse. Scale bar, 100 μm. **E** Representative images and the percentage of EGFP-labelled neurons in the eLPB ChAT-positvie populations. Scale bar, 100 μm. **F** Representative images and the percentage of surrounded PKCδ-positive (PKCδ^+^) neurons in the lCeA and ovBNST. Scale bar, 100 μm. **G** Schematic diagram of the CTB-555 injection in the lCeA, the viral injection sites in the lCeA and CTB-555-labeled and ChAT that co-expressed within the eLPB of wild-type (WT) mouse. Scale bar, 100 μm. **H** Schematic diagram of the CTB-555 injection in the ovBNST, the viral injection sites in the ovBNST and CTB-555-labeled and ChAT that co-expressed within the eLPB of WT mouse. Scale bar, 100 μm.

We conducted separate retrograde and anterograde tracer experiments in different batches of mice in the current study. Consistent with our previous study 14, we observed a widespread distribution of ChAT^+^ neurons in the eLPB^ChAT^ along the anterior-posterior brain axis in naïve mice (Figures 1A, 1B and 1C). Here, employing the anterograde (Figures 1D, 1E and 1F) and retrograde (Figures 1G and 1H) virus tracing, we found that eLPB^ChAT^ neurons predominantly projected to lateral portion of CeA (lCeA) and the oval portion of BNST (ovBNST). Furthermore, approximately 75% and 52% of eLPB^ChAT^ terminals were distributed around PKCδ-positive (PKCδ^+^) neurons in lCeA and ovBNST respectively, forming the eLPB^ChAT^-lCeA^PKCδ^ pathway and eLPB^ChAT^-ovBNST^PKCδ^ pathway.

**Figure 2:**
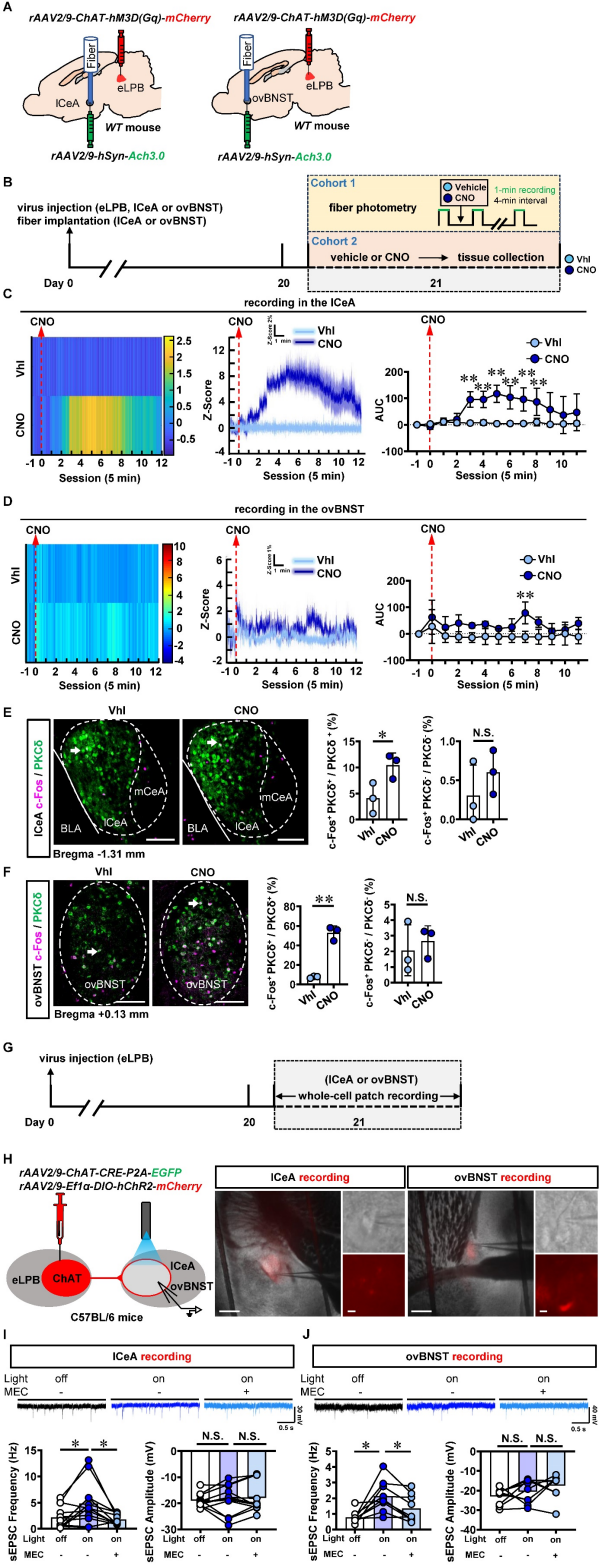
** Functional innervation of the eLPB^ChAT^-lCeA^PKCδ^ and eLPB^ChAT^-ovBNST^PKCδ^ pathways in naïve male mice. A** Schematic diagram of *rAAV*2/9*-ChAT-hM3D(Gq)-mCherry* injection into eLPB and fiber implantation in lCeA or ovBNST in WT mouse. **B** Experimental design and timeline in naïve male mice. **C** Heatmap (left), quantification (middle) and AUC (right) of Z-Score of Ach3.0 fluorescence in the lCeA. Two-way ANOVA with Sidak's multiple comparisons test. n = 5 mice per group. F _(12, 104)_ = 4.730, *p* < 0.0001; t = 4.545, *p (15 min) = 0.0002; t = 4.482, *p (20 min) = 0.0002; t = 5.780, **p (25 min) < 0.0001; t = 5.069, *p (30 min) < 0.0001; t = 4.675, *p (35 min) = 0.0001; t = 3.900, *p (40 min) = 0.0022. **D** Heatmap (left), quantification (middle) and AUC (right) of Z-Score of Ach3.0 fluorescence in the ovBNST. Two-way ANOVA with Sidak's multiple comparisons test. n = 3 mice per group. F _(12, 52)_ = 0.7841, *p* = 0.6640; t = 3.613, **p (35 min) = 0.0088.** E** The percentage of c-Fos-positive (c-Fos^+^) neurons in the lCeA PKCδ^+^ and PKCδ-negative (PKCδ^-^) neurons. Two-tailed unpaired t test. n = 3 mice per group. c-Fos^+^ PKCδ^+^, t _(4)_ = 2.925, *p* = 0.043; c-Fos^+^ PKCδ^-^, t _(4)_ = 1.076, *p* = 0.3426. Scale bar, 100 μm. **F** The percentage of c-Fos^+^ neurons in the ovBNST PKCδ^+^ and PKCδ^-^ neurons. Two-tailed unpaired t test. n = 3 mice per group. c-Fos^+^ PKCδ^+^, t _(4)_ = 10.89, *p* = 0.0004; c-Fos^+^ PKCδ^-^, t _(4)_ = 0.5378, *p* = 0.6192. Scale bar, 100 μm.** G** Experimental design and timeline of whole-cell path recording.** H** Schematic diagram of the viral transfection in mice and representative images of patch-clamp recording on the lCeA or ovBNST. Scale bar, 200 μm/5 μm. **I** Representative traces and statistics data of the lCeA neurons sEPSC. n = 12 cells from 6 mice. The one-way analysis of variance (ANOVA) with Tukey post-test. Frequency, F _(1.440, 15.84)_ = 7.384, *p* = 0.0093; *p = 0.0316 off vs on; *p = 0.0365 on vs on-MEC; Amplitude, F _(1.825, 20.07)_ = 0.4156, *p* = 0.6473; *p* = 0.9690 off vs on; *p* = 0.7725 on vs on-MEC. **J** Representative traces and statistics data of the ovBNST neurons sEPSC. n = 9 cells from 6 mice. The one-way analysis of variance (ANOVA) with Tukey post-test. Frequency, F _(1.486, 11.89)_ = 9.790, *p* = 0.0049; * p = 0.0156 off vs on; *p = 0.0115 on vs on-MEC; Amplitude, F _(1.859, 14.87)_ = 2.762, *p* = 0.0983; *p* = 0.5846 off vs on;* p* = 0.4936 on vs on-MEC. Vhl, vehicle; CNO, clozapine-N-oxide. *, *p* < 0.05, ** *p* < 0.01 vs Vhl.

Since cholinergic neurons have the potential to act as interneurons for local regulation 25 or innervating other nuclei through long projections 26, 27 in the brain, we explored the physiological innervation of eLPB^ChAT^ neurons on PKCδ^+^ neurons in lCeA and ovBNST. First, to observe the real-time Ach signals from eLPB^ChAT^ terminals within the lCeA and ovBNST, DREADDs method and fiber photometry were employed in male naïve mice (Figures 2A, 2B and S1A). As shown in Figure S1B, systemic administration of CNO efficiently activates eLPB^ChAT^ neurons. In parallel, the Ach release was significantly increased in lCeA from 15 to 40 min (Figure 2C), and in ovBNST at 35 min following CNO injection (Figure 2D) in free-moving male mice compared to vehicle controls. In another set of identical mouse models, we collected brain tissue at 35 min after CNO injection and found that both lCeA^PKCδ^ neurons (Figure 2E) and ovBNST^PKCδ^ neurons (Figure 2F) were evoked by activating eLPB^ChAT^ neurons, as indicated by increased c-Fos^+^ PKCδ^+^ neurons. To further characterize the potential postsynaptic elements involved in the eLPB^ChAT^-lCeA/ovBNST pathway, we employed optogenetic activation strategies combined with patch-clamp recordings on acutely prepared slices (Figures 2G and 2H). Blue light (473 nm) was utilized to activate the eLPB^ChAT^ terminals within either lCeA or ovBNST. As shown in Figures 2I and 2J, the frequency of sEPSC in the lCeA and ovBNST neurons was increased when activating LPB^ChAT^ terminals. Inhibition of nAChRs with mecamylamine incubation during optogenetic activation of eLPB^ChAT^ terminals resulted in a blockade of the triggered sEPSC frequency in slices of lCeA and ovBNST. Notably, no significant differences were observed in the amplitude of sEPSC between the lCeA and ovBNST neurons throughout the entire process. Collectively, these findings suggest that eLPB^ChAT^ neurons positively modulate lCeA^PKCδ^ and ovBNST^PKCδ^ neurons, at least partially through synaptic elements of presynaptic Ach release and postsynaptic nAChRs.

### Inhibiting eLPB^ChAT^-lCeA^PKCδ^ pathway alleviates anxiety-like behaviors in METH-withdrawn male mice

**Figure 3:**
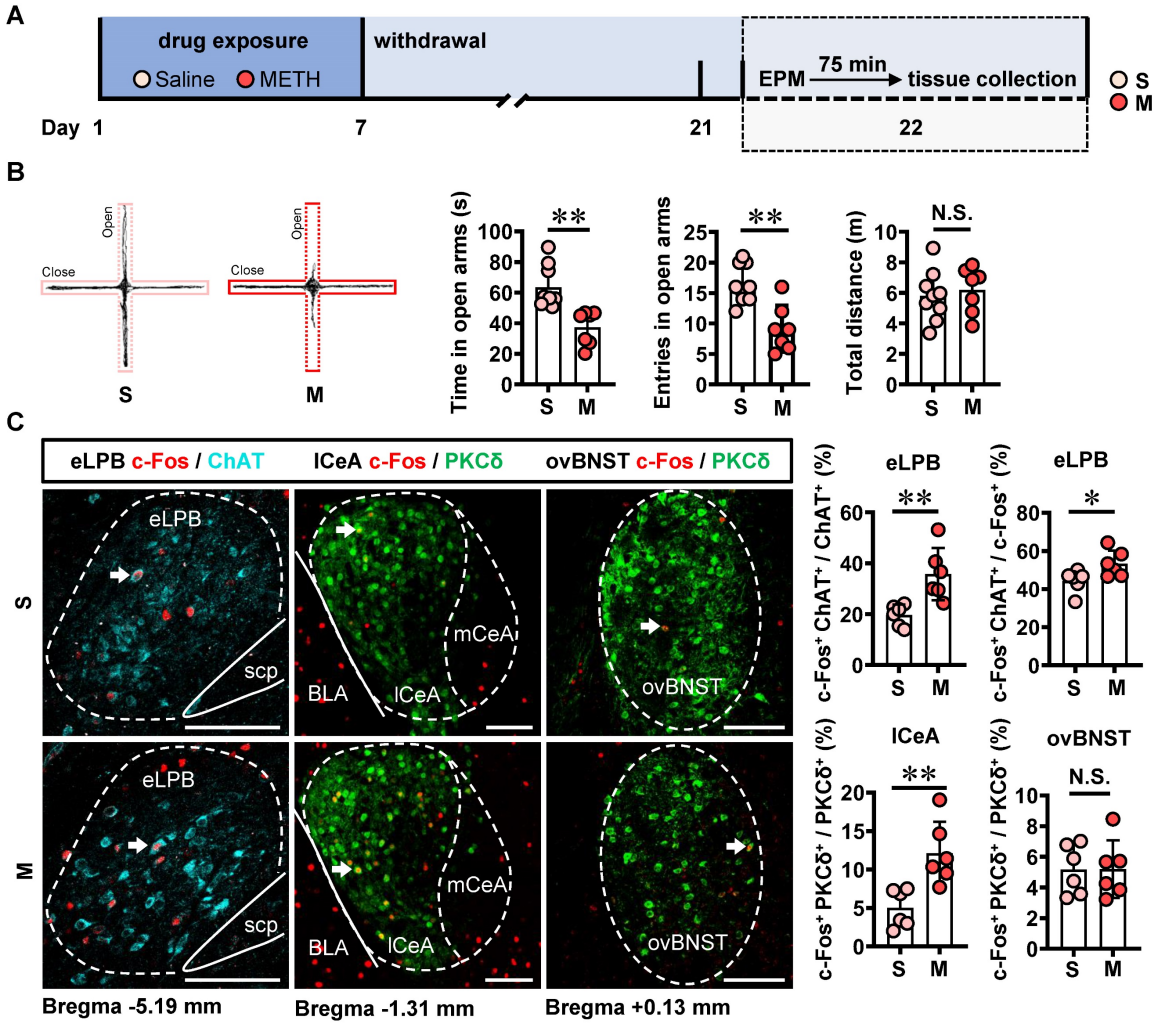
** METH withdrawal enhances anxiety-like behaviors and triggers excitability of eLPB^ChAT^ and lCeA^PKCδ^ neurons in male mice. A** Experimental design and timeline. **B** EPM test. Two-tailed unpaired t test. S group, n = 9 mice; M group, n = 7 mice. Time in open arms, t _(14)_ = 4.060, *p* = 0.0012. Entries into open arms, t _(14)_ = 4.194, *p* = 0.0009. Total distance, t _(14)_ = 0.4946, *p* = 0.6285. **C** Representative images and the percentage of c-Fos^+^ neurons in the eLPB^ChAT^, lCeA^PKCδ^ and ovBNST^PKCδ^ neurons. Two-tailed unpaired t test. n = 6 mice per group. eLPB (c-Fos^+^ ChAT^+^ / ChAT^+^%), t _(10)_ = 3.539, *p* = 0.0054; eLPB (c-Fos^+^ ChAT^+^ / c-Fos^ +^%), t _(10)_ = 2.413, *p* = 0.0365; lCeA, t _(10)_ = 3.636, *p* = 0.0046; ovBNST, t _(10)_ = 0.02581, *p* = 0.9799.

The involvement of LPB-CeA and LPB-BNST pathways in anxiety has been previously reported 15, 16, 28, 29. Here, mice were subjected to daily intraperitoneal injections of METH or saline for 7 consecutive days, followed by a 14-day withdrawal period. Anxiety-like behaviors were assessed using EPM on Day 22 (Figure 3A).

In comparison with the saline-withdrawn mice, METH-withdrawn mice exhibited anxiety-like behaviors characterized by reduced time spent and fewer entries into the open arms (Figure 3B). Additionally, both eLPB^ChAT^ neurons and lCeA^PKCδ^ neurons, but not ovBNST^PKCδ^ neurons, showed significantly activation in METH-withdrawn male mice (Figure 3C).

**Figure 4:**
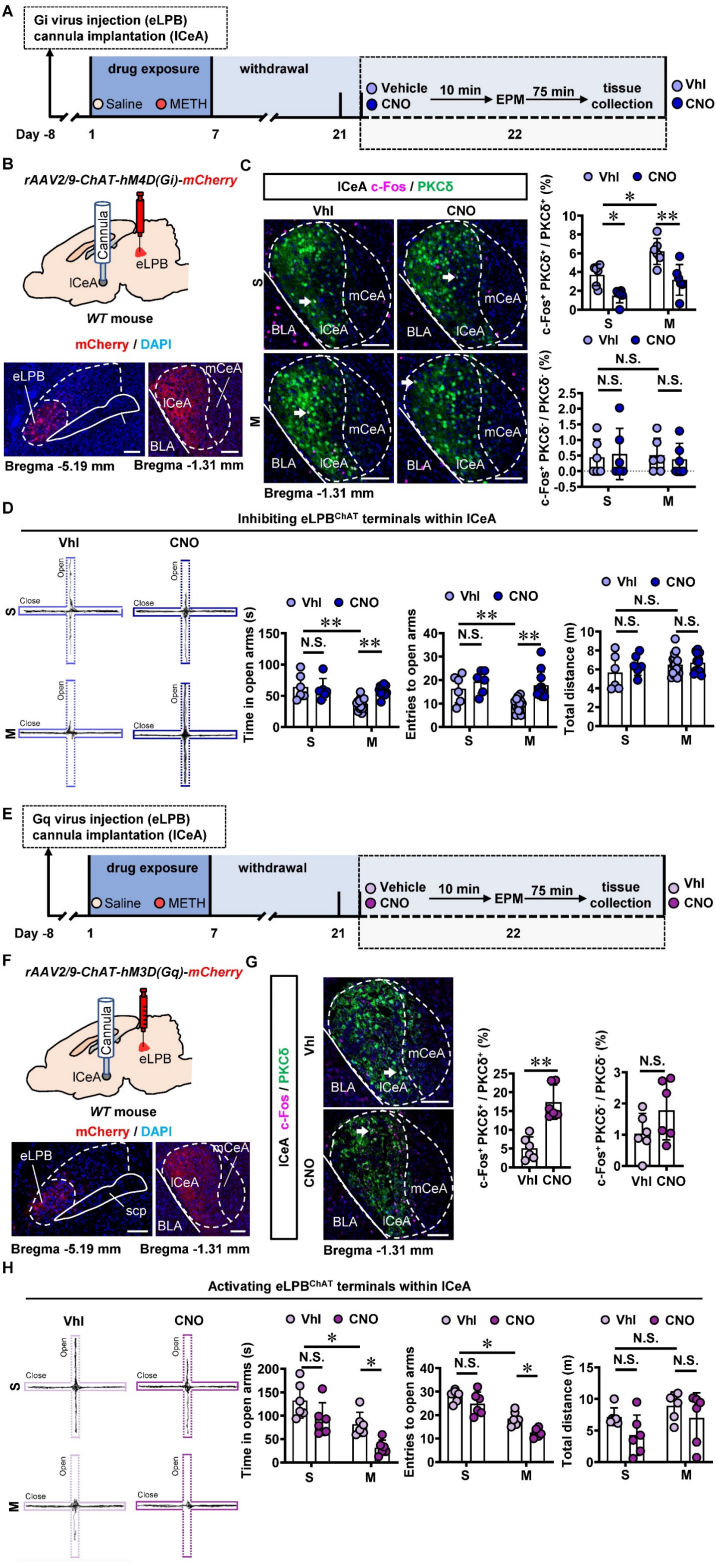
** Inhibiting eLPB^ChAT^-lCeA^PKCδ^ pathway alleviates the anxiety-like behaviors in METH-withdrawn male mice. A** Experimental design and timeline of Gi virus in the eLPB. **B** Schematics and representative images of *rAAV2/9-ChAT-hM4D(Gi)-mCherry* injection in the eLPB, cannula implantation in the lCeA, and mCherry+ axon terminals in the lCeA of WT mouse. **C** Representative images and the percentage of c-Fos^+^ neurons in the lCeA PKCδ^+^ and PKCδ^-^ neurons. Two-way ANOVA with Sidak's multiple comparisons test. Upper, n = 6 mice per group. F _(1, 20)_ = 0.6286, *p* = 0.4372; S-CNO group, t = 3.024, *p* = 0.0395 vs S-Vhl group; M-CNO group, t = 4.145, *p* = 0.0030 vs M-Vhl group; M-Vhl group, t = 3.405, *p* = 0.0167 vs S-Vhl group. Lower, n = 6 mice per group. F _(1, 20)_ = 0.2019, *p* = 0.6580; S-CNO group, t = 0.2862, *p* = 0.9999 vs S-Vhl group; M-CNO group, t = 0.3492, *p* = 0.9996 vs M-Vhl group; M-Vhl group, t = 0.1561, *p* > 0.9999 vs S-Vhl group. **D** EPM test. Two-way ANOVA with Sidak's multiple comparisons test. S-Vhl group, n = 6 mice; S-CNO group, n = 6 mice; M-Vhl group, n = 16 mice; M-CNO group, n = 12 mice. Time in open arms, F _(1, 36)_ = 7.915, *p* = 0.0079; S-CNO group, t = 0.5496, *p* = 0.9950 vs S-Vhl group; M-CNO group, t = 4.269, *p* = 0.0008 vs M-Vhl group; M-Vhl group, t = 4.552, *p* = 0.0004 vs S-Vhl group. Entries into open arms, F _(1, 36)_ = 2.688, *p* = 0.1098; S-CNO group, t = 1.132, *p* = 0.8423 vs S-Vhl group; M-CNO group, t = 4.684, *p* = 0.0002 vs M-Vhl group; M-Vhl group, t = 3.054, *p* = 0.0251 vs S-Vhl group. Total distance, F _(1, 36)_ = 0.5195, *p* = 0.4757; S-CNO group, t = 1.138, *p* = 0.8395 vs S-Vhl group; M-CNO group, t = 0.4135, *p* = 0.9990 vs M-Vhl group; M-Vhl group, t = 1.446, *p* = 0.6409 vs S-Vhl group. **E** Experimental design and timeline of Gq virus in the eLPB. **F** Schematics and representative images of *rAAV2/9-ChAT-hM3D(Gq)-mCherry* injection in the eLPB, cannula implantation in the lCeA, and mCherry+ axon terminals in the lCeA of WT mouse. **G** Representative images and the percentage of the percentage of c-Fos^+^ neurons in and lCeA PKCδ^+^ and PKCδ^-^ neurons. Two-tailed unpaired t test. n = 6 mice per group. c-Fos^+^ PKCδ^+^ / PKCδ^+^, t _(10)_ = 5.514, *p* = 0.0003; c-Fos^+^ PKCδ^-^ / PKCδ^-^, t _(10)_ = 1.595, *p* = 0.1417. **H** EPM test. Two-way ANOVA with Sidak's multiple comparisons test. n = 6 mice per group. Time in open arms, F _(1, 20)_ = 0.1872, *p* = 0.6699; S-CNO group, t = 2.313, *p* = 0.1745 vs S-Vhl group; M-CNO group, t = 2.925, *p* = 0.0492 vs M-Vhl group; M-Vhl group, t = 2.957, *p* = 0.0458 vs S-Vhl group. Entries in open arms, F _(1, 20)_ = 0.9668, *p* = 0.3372; S-CNO group, t = 1.738, *p* = 0.4599 vs S-Vhl group; M-CNO group, t = 3.129, *p* = 0.0313 vs M-Vhl group; M-Vhl group, t = 5.041, t = 0.0004 vs S-Vhl group. Total distance, F _(1, 20)_ = 0.1705, *p* = 0.6841; S-CNO group, t = 1.764, *p* = 0.4430 vs S-Vhl group; M-CNO group, t = 1.180, *p* = 0.8244 vs M-Vhl group; M-Vhl group, t = 1.063, *p* = 0.8827 vs S-Vhl group. Vhl, vehicle; CNO, clozapine-N-oxide; S, saline; M, methamphetamine; N.S., *p* > 0.05, *, *p* < 0.05, **, *p* < 0.01 vs S or Vhl or CNO. Scale bar, 100 μm.

To investigate the role of eLPB^ChAT^-lCeA pathway in METH withdrawal-induced anxiety-like behavior, *rAAV2/9-ChAT-hM4D(Gi)-mCherry* virus was injected into the eLPB, and cannulas were placed in the lCeA. 10 min before EPM test, CNO or vehicle was locally infused through the cannula to selectively inhibit eLPB^ChAT^ terminals within the lCeA or not (Figures 4A and 4B). Results showed that inhibiting eLPB^ChAT^-lCeA^PKCδ^ pathway decreased the activity of PKCδ^+^ neurons without affecting PKCδ^-^ neurons of the lCeA in both METH-withdrawn and control mice (Figure 4C). Additionally, inhibition of eLPB^ChAT^-lCeA^PKCδ^ pathway efficiently attenuated anxiety-like behaviors specifically in METH-withdrawn male mice, while having no impact on related behaviors in controls (Figure 4D). Conversely, when we activated eLPB^ChAT^ terminals within the lCeA using similar viral tools (Figures 4E and 4F), there was an increased activation of PKCδ^+^ neurons but not PKCδ^-^ neurons in the lCeA (Figure 4G). As depicted in Figure 4H, activating eLPB^ChAT^-lCeA^PKCδ^ pathway induced heightened anxiety-like behavior specifically in METH-withdrawn mice, while having no influence on such behavior in control mice.

Taken together, these findings suggest that the eLPB^ChAT^-lCeA^PKCδ^ pathway is involved in encoding METH withdrawal anxiety, and has no influence on anxiety-like behavior in saline withdrawal mice.

### Activating eLPB^ChAT^-ovBNST^PKCδ^ pathway suppresses METH-primed reinstatement of CPP in male METH-exposed mice

**Figure 5:**
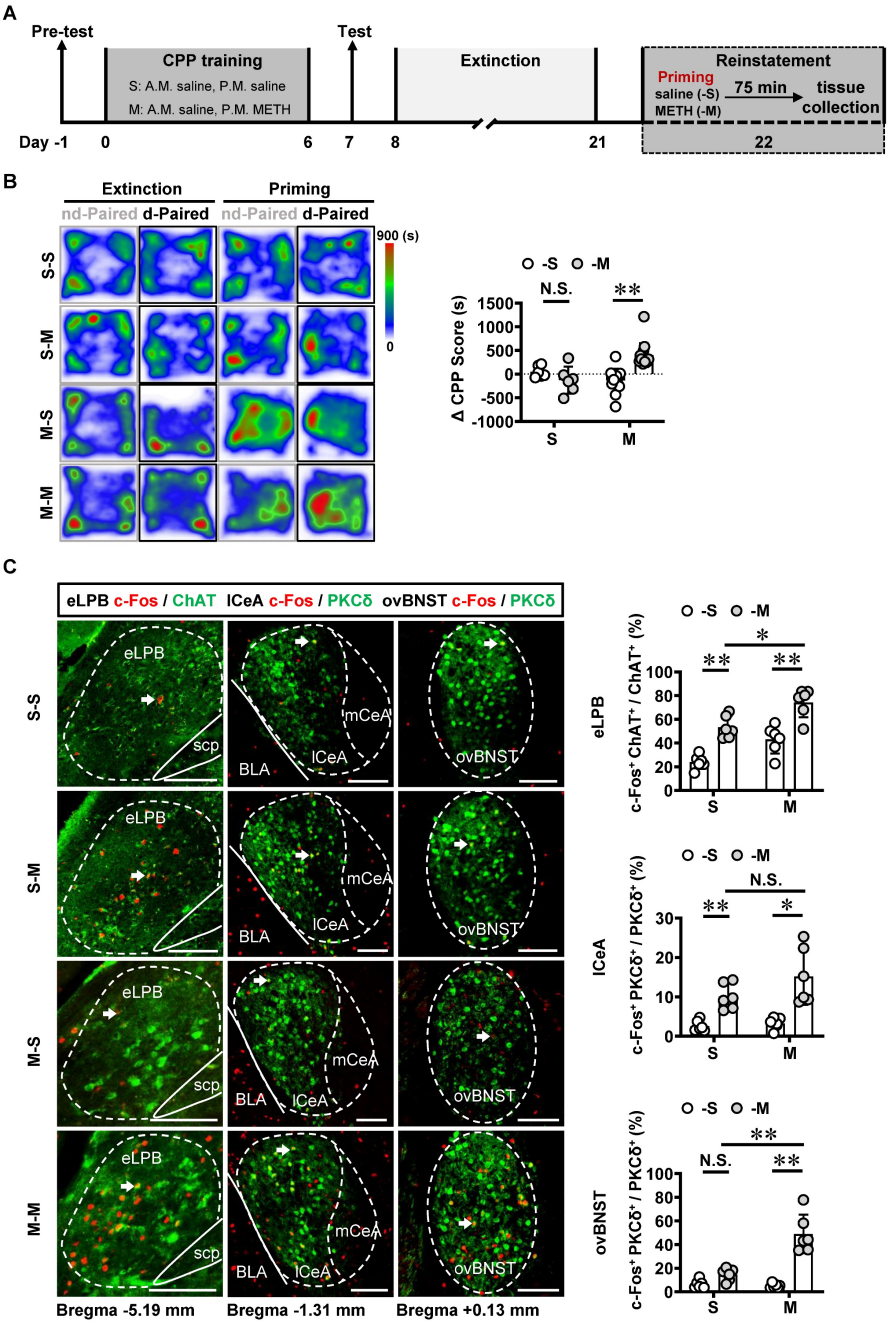
** METH-primed reinstatement of CPP triggers excitability of the eLPB^ChAT^, lCeA^PKCδ^ and ovBNST^PKCδ^ neurons in male mice. A** Experimental design and timeline. **B** Heatmap of spent duration by mice in CPP apparatus and ∆CPP score. Two-way ANOVA with Sidak's multiple comparisons test. S group, n = 6 mice per group; M group, n = 14 mice per group. F _(1, 36)_ = 18.16, *p* = 0.0001; S-M group, t = 1.267, *p* = 0.7630 vs S-S group; M-M group, t = 5.846, *p* < 0.0001 vs M-S group. **C** Representative images and the percentage of c-Fos^+^ neurons in the eLPB^ChAT^, lCeA^PKCδ^ and ovBNST^PKCδ^ neurons. Two-way ANOVA with Sidak's multiple comparisons test. n = 6 mice per group. eLPB, F _(1, 20)_ = 0.04656, *p* = 0.8313; S-M group, t = 4.967, *p* = 0.0004 vs S-S group; M-M group, t = 5.272, *p* = 0.0002 vs M-S group; M-M group, t = 3.527, *p* = 0.0126 vs S-M group. lCeA, F _(1, 20)_ = 1.737, *p* = 0.2024; S-M group, t = 3.183, *p* = 0.0277 vs S-S group; M-M group, t = 5.047, *p* = 0.0004 vs M-S group; M-M group, t = 2.149, *p* = 0.2367 vs S-M group. ovBNST, F _(1, 20)_ = 24.78, *p* < 0.0001; S-M group, t = 1.587, *p* = 0.5608 vs S-S group; M-M group, t = 8.627, *p* < 0.0001 vs M-S group; M-M group, t = 6.764, *p* < 0.0001 vs S-M group. Scale bar, 100 μm. N.S., *p* > 0.05, *, *p* < 0.05, **, *p* < 0.01 vs S-S or M-S or Vhl or CNO.

Recently, emerging evidence demonstrate that PKCδ^+^ GABAergic neurons might involve in encoding relapse to drugs 19, 30. The METH-primed reinstatement of CPP procedure was established in male mice (Figure 5A). Our findings revealed that a single METH challenge (0.5 mg/kg) effectively induced the reinstatement of CPP in METH-exposed mice rather than those exposed to saline (Figures 5B and S2A). For the METH extinction mice, METH challenge resulted in heightened activity levels in eLPB^ChAT^, lCeA^PKCδ^ and ovBNST^PKCδ^ neurons (Figure 5C). Further, METH priming induced a more pronounced increase in eLPB^ChAT^ and ovBNST^PKCδ^ neurons, with no discernible impact on lCeA^PKCδ^ neurons in METH-exposed mice compared to saline-exposed mice. These findings suggest that ovBNST^PKCδ^ neurons play a more prominent role in mediating METH-primed reinstatement of METH CPP.

**Figure 6:**
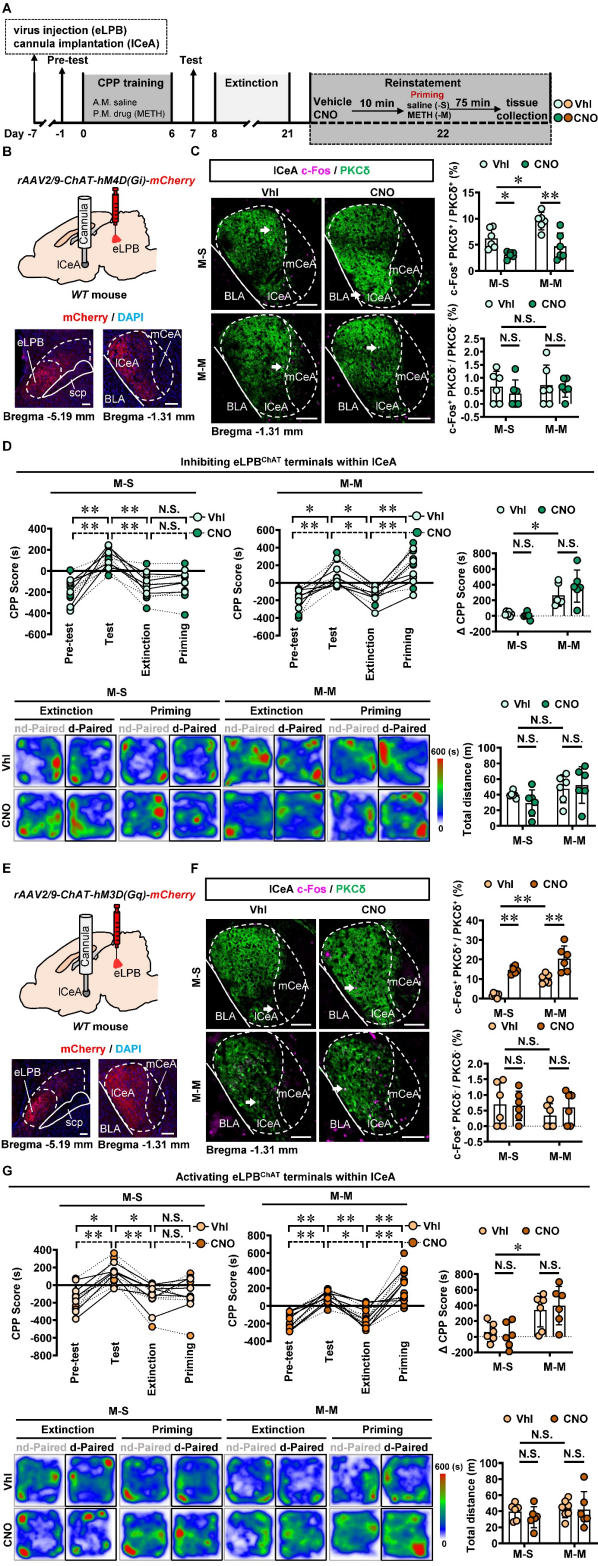
** Manipulation of eLPB^ChAT^-lCeA^PKCδ^ pathway had no influence on METH-primed reinstatement of CPP in mice. A** Experimental design and timeline. **B** Schematics and representative images of *rAAV2/9-ChAT-hM4D(Gi)-mCherry* injection in the eLPB, cannula implantation into the lCeA, and mCherry+ axon terminals in the lCeA of WT mouse. **C** Representative images and the percentage of c-Fos^+^ neurons in PKCδ^+^ and PKCδ^-^ neurons of lCeA. Two-way ANOVA with Sidak's multiple comparisons test. Upper, n = 6 mice per group. F _(1, 20)_ = 1.300, *p* = 0.2676; M-S-CNO group, t = 2.956, *p* = 0.0459 vs M-S-Vhl group; M-M-CNO group, t = 4.569, *p* = 0.0011 vs M-M-Vhl group; M-M-Vhl group, t = 3.141, *p* = 0.0305 vs M-S-Vhl group. Lower, n = 6 mice per group. F _(1, 20)_ = 0.1319, *p* = 0.7203; M-S-CNO group, t = 0.8018, *p* = 0.9664 vs M-S-Vhl group; M-M-CNO group, t = 0.2883, *p* = 0.9999 vs M-M-Vhl group; M-M-Vhl group, t = 0.1643, *p* > 0.9999 vs M-S-Vhl group. Scale bar, 100 μm. **D** CPP scores during the pre-test, CPP test, extinction and priming in M-S and M-M group and ∆CPP score (priming CPP score minus extinction CPP score). Two-way ANOVA with Sidak's multiple comparisons test. CPP scores, M-S group, n = 6 mice per group. F _(3, 40)_ = 0.2925, *p* = 0.8305; M-S-Vhl group priming, t = 0.3979, *p* = 0.9992 vs extinction; M-S-CNO group priming, t = 0.03663, *p* > 0.9999 vs extinction; M-M group, n = 6 mice per group. F _(3, 40)_ = 0.9096, *p* = 0.4450; M-M-Vhl group priming, t = 3.380, *p* = 0.0097 vs extinction; M-M-CNO group priming, t = 4.884, *p* = 0.0001 vs extinction. ∆CPP score, n = 6 mice per group. F _(1, 20)_ = 1.919, *p* = 0.1812; M-S-CNO group, t = 0.3304, *p* = 0.9997 vs M-S-Vhl group; M-M-CNO group, t = 1.629, *p* = 0.5324 vs M-M-Vhl group; M-M-Vhl group, t = 3.224, *p* = 0.0253 vs M-S-Vhl group. Heatmap of spent duration by mice in CPP apparatus and total distance traveled in CPP apparatus during priming test. Two-way ANOVA with Sidak's multiple comparisons test. n = 6 mice per group. F _(1, 20)_ = 1.169, *p* = 0.2924; M-S-CNO group, t = 1.068, *p* = 0.8805 vs M-S-Vhl group; M-M-CNO group, t = 0.4610, *p* = 0.9982 vs M-M-Vhl group; M-M-Vhl group, t = 0.7778, *p* = 0.9710 vs M-S-Vhl group. **E** Schematics and representative images of *rAAV2/9-ChAT-hM3D(Gq)-mCherry* injection in the eLPB, cannula implantation into the lCeA, and mCherry+ axon terminals in the lCeA of WT mouse. **F** Representative images and the percentage of c-Fos^+^ neurons in the lCeA PKCδ^+^ and PKCδ^-^ neurons. Two-way ANOVA with Sidak's multiple comparisons test. Upper, n = 6 mice per group. F _(1, 20)_ = 0.7879, *p* = 0.3853; M-S-CNO group, t = 6.110, *p* < 0.0001 vs M-S-Vhl group; M-M-CNO group, t = 4.855, *p* = 0.0006 vs M-M-Vhl group; M-M-Vhl group, t = 3.940, *p* = 0.0048 vs M-S-Vhl group. Lower, n = 6 mice per group. F _(1, 20)_ = 0.5010, *p* = 0.4872; M-S-CNO group, t = 0.1483, *p* > 0.9999 vs M-S-Vhl group; M-M-CNO group, t = 0.8527, *p* = 0.9552 vs M-M-Vhl group; M-M-Vhl group, t = 1.181, *p* = 0.8242 vs M-S-Vhl group. Scale bar, 100 μm.** G** CPP scores during the pre-test, CPP test, extinction and priming in M-S and M-M group and ∆CPP score. Two-way ANOVA with Sidak's multiple comparisons test. CPP scores, M-S-Vhl, n = 6 mice; M-S-CNO group, n = 6 mice; M-M-Vhl group, n = 7 mice; M-M-CNO group, n = 6 mice; M-S group, F _(3, 40)_ = 0.2600, *p* = 0.8537; M-S-Vhl group priming, t = 0.6514, *p* = 0.9875 vs extinction; M-S-CNO group priming, t = 0.2366, *p* > 0.9999 vs extinction; M-M group, F_(3, 44)_ = 0.1465, *p* = 0.9314; M-M-Vhl group priming, t = 5.055, *p* < 0.0001 vs extinction; M-M-CNO group priming, t = 5.445, *p* < 0.0001 vs extinction. ∆CPP score, M-S-Vhl group, n = 6 mice; M-S-CNO group, n = 6 mice; M-M-Vhl group, n = 7 mice. M-S-CNO group, n = 6 mice. F _(1, 21)_ = 0.3640, *p* = 0.5528; M-S-CNO group, t = 0.3362, *p* = 0.9997 vs M-S-Vhl group; M-M-CNO group, t = 0.5205, *p* = 0.9964 vs M-M-Vhl group; M-M-Vhl group, t = 2.633, *p* = 0.0894 vs M-S-Vhl group. Heatmap of spent duration by mice in CPP apparatus and total distance traveled in CPP apparatus during priming test. Two-way ANOVA with Sidak's multiple comparisons test. M-S-Vhl group, n = 6 mice; M-S-CNO group, n = 6 mice; M-M-Vhl group, n = 7 mice; M-S-CNO group, n = 6 mice. F _(1, 21)_ = 0.2734, *p* = 0.6066; M-S-CNO group, t = 0.8464, *p* = 0.9565 vs M-S-Vhl group; M-M-CNO group, t = 0.1249, *p* > 0.9999 vs M-M-Vhl group; M-M-Vhl group, t = 0.4007, *p* = 0.9992 vs M-S-Vhl group. Vhl, vehicle; CNO, clozapine-N-oxide; M-S, saline-primed reinstatement test following METH CPP extinction training; M-M, METH-primed reinstatement test following METH CPP extinction training; N.S., *p* > 0.05, *, *p* < 0.05, **, *p* < 0.01.

Based on the neuronal activation phenotypes observed in METH-primed mice in the present study, we speculate that precise inhibition of the eLPB^ChAT^-lCeA^PKCδ^ or eLPB^ChAT^-ovBNST^PKCδ^ pathway may attenuate the METH-primed reinstatement of CPP in METH-exposed mice. As shown in Figures 6A-6C, utilizing DREADDs methodology, we successfully achieved inhibition of eLPB^ChAT^ terminals in the lCeA^PKCδ^ in METH-exposed mice. Contrary to our initial expectations, inhibiting the eLPB^ChAT^-lCeA^PKCδ^ pathway did not exert any influence on METH-primed reinstatement of CPP in mice (Figure 6D). Furthermore, there were no significant differences in ∆CPP score between the vehicle-treated and CNO-treated groups in saline-challenged METH-exposed mice (Figure 6D), suggesting that inhibiting eLPB^ChAT^ terminals in lCeA^PKCδ^ without administrating METH challenge cannot facilitate the reinstatement of CPP in mice. Previously, our research showed that activating eLPB^ChAT^ neurons or CeA-projecting eLPB^ChAT^ neurons, rather than suppressing them, could prevent METH-primed reinstatement of CPP in mice 14. Therefore, we conducted local activation of eLPB^ChAT^ terminals in the lCeA^PKCδ^ in METH-exposed mice (Figures 6E and 6F). Unexpectedly, activating the eLPB^ChAT^-lCeA^PKCδ^ pathway also failed to elicit any impact on METH-primed reinstatement of METH CPP in mice (Figure 6G), indicating that this pathway does not regulate METH-primed reinstatement behavior. These findings raise questions regarding other potential pathways involving eLPB^ChAT^ neurons responsible for modulating METH-primed reinstatement.

**Figure 7:**
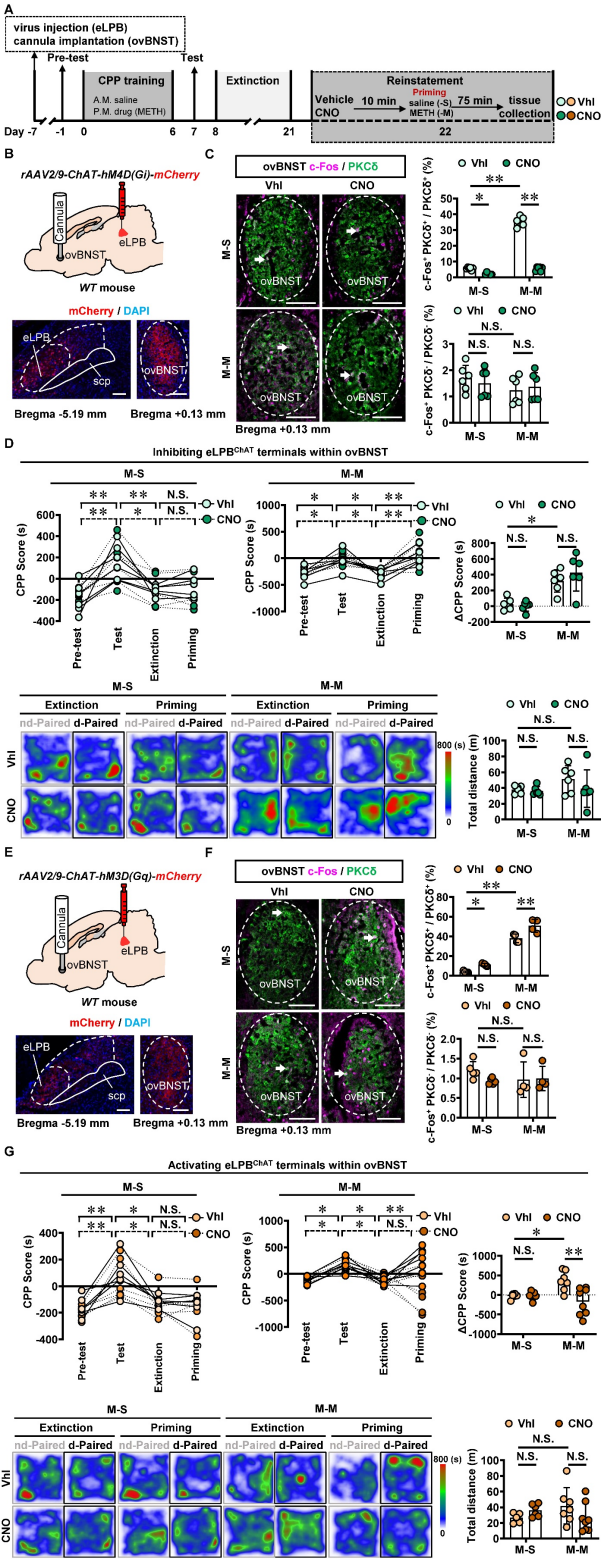
** Activating eLPB^ChAT^-ovBNST^PKCδ^ pathway suppresses METH-primed reinstatement of CPP in male METH-exposed mice. A** Experimental design and timeline. **B** Schematics and representative images of *rAAV2/9-ChAT-hM4D(Gi)-mCherry* injection in the eLPB, cannula implantation into the ovBNST, and mCherry+ axon terminals in the ovBNST of WT mouse. **C** Representative images and the percentage of c-Fos^+^ neurons in PKCδ^+^ and PKCδ^-^ neurons of the ovBNST. Two-way ANOVA with Sidak's multiple comparisons test. Upper, n = 6 mice per group. F _(1, 20)_ = 351.3, *p* < 0.0001; M-S-CNO group, t = 3.564, *p* = 0.0116 vs M-S-Vhl group; M-M-CNO group, t = 30.07, *p* < 0.0001 vs M-M-Vhl group; M-M-Vhl group, t = 29.87, *p* < 0.0001 vs M-S-Vhl group. Lower, n = 6 mice per group. F _(1, 20)_ = 0.6128, *p* = 0.4429; M-S-CNO group, t = 0.6598, *p* = 0.9873 vs M-S-Vhl group; M-M-CNO group, t = 0.4473, *p* = 0.9984 vs M-M-Vhl group; M-M-Vhl group, t = 1.529, *p* = 0.6009 vs M-S-Vhl group. Scale bar, 100 μm. **D** CPP scores during the pre-test, CPP test, extinction and priming in M-S and M-M group and ∆CPP score. Two-way ANOVA with Sidak's multiple comparisons test. CPP scores, n = 6 mice per group. M-S group, F _(3, 40)_ = 0.1050, *p* = 0.9567; M-S-Vhl group priming, t = 0.3309, *p* = 0.9997 vs extinction; M-S-CNO group priming, t = 0.003933, *p* > 0.9999 vs extinction; M-M group, n = 6 mice per group. F _(3, 40)_ = 0.3636, *p* = 0.7796; M-M-Vhl group priming, t = 3.698, *p* = 0.0039 vs extinction; M-M-CNO group priming, t = 4.924, *p* < 0.0001 vs extinction. ∆CPP score, n = 6 mice per group. F _(1, 20)_ = 1.261, *p* = 0.2748; M-S-CNO group, t = 0.3214, *p* = 0.9998 vs M-S-Vhl group; M-M-CNO group, t = 1.267, *p* = 0.7745 vs M-M-Vhl group; M-M-Vhl group, t = 3.502, *p* = 0.0134 vs M-S-Vhl group. Heatmap of spent duration by mice in CPP apparatus and total distance traveled in CPP apparatus during priming test. Two-way ANOVA with Sidak's multiple comparisons test. n = 6 mice per group. F _(1, 20)_ = 0.6880, *p* = 0.4167; M-S-CNO group, t = 0.2092, *p* > 0.9999 vs M-S-Vhl group; M-M-CNO group, t = 1.382, *p* = 0.7007 vs M-M-Vhl group; M-M-Vhl group, t = 1.515, *p* = 0.6106 vs M-S-Vhl group. **E** Schematics and representative images of *rAAV2/9-ChAT-hM3D(Gq)-mCherry* injection in the eLPB, cannula implantation in the ovBNST, and mCherry+ axon terminals in the ovBNST of WT mouse.** F** Representative images and the percentage of c-Fos^+^ neurons in the ovBNST PKCδ^+^ and PKCδ^-^ neurons. Two-way ANOVA with Sidak's multiple comparisons test. Upper, M-S group, n = 5 mice per group; M-M group, n = 4 mice per group. F _(1, 14)_ = 2.226, *p* = 0.1579; M-S-CNO group, t = 3.091, *p* = 0.0469 vs M-S-Vhl group; M-M-CNO group, t = 4.766, *p* = 0.0018 vs M-M-Vhl group; M-M-Vhl group, t = 13.42, *p* < 0.0001 vs M-S-Vhl group. Lower, M-S group, n = 5 mice per group; M-M group, n = 4 mice per group. F _(1, 14)_ = 1.312, *p* = 0.2713; M-S-CNO group, t = 1.562, *p* = 0.5972 vs M-S-Vhl group; M-M-CNO group, t = 0.1395, *p* > 0.9999 vs M-M-Vhl group; M-M-Vhl group, t = 1.270, *p* = 0.7828 vs M-S-Vhl group. Scale bar, 100 μm. **G** CPP scores during the pre-test, CPP test, extinction and priming in M-S and M-M group and ∆CPP score. Two-way ANOVA with Sidak's multiple comparisons test. CPP scores, M-S-Vhl group, n = 6 mice; M-S-CNO group, n = 6 mice; M-M-Vhl group, n = 7 mice; M-M-CNO group, n = 8 mice. M-S group, F _(3, 40)_ = 0.08858, *p* = 0.9659; M-S-Vhl group priming, t = 0.4327, *p* = 0.9986 vs extinction; M-S-CNO group priming, t = 0.1847, *p* > 0.9999 vs extinction; M-M group, F _(3, 52)_ = 8.126, *p* = 0.0002; M-M-Vhl group priming, t = 3.769, *p* = 0.0025 vs extinction; M-M-CNO group priming, t = 1.952, *p* = 0.2937 vs extinction. ∆CPP score, M-S-Vhl group, n = 6 mice; M-S-CNO group, n = 6 mice; M-M-Vhl group, n = 7 mice; M-M-CNO group, n = 8 mice. F _(1, 23)_ = 8.648, *p* = 0.0073; M-S-CNO group, t = 0.1133, *p* > 0.9999 vs M-S-Vhl group; M-M-CNO group, t = 4.279, *p* = 0.0017 vs M-M-Vhl group; M-M-Vhl group, t = 2.887, *p* = 0.0489 vs M-S-Vhl group. Heatmap of spent duration by mice in CPP apparatus and total distance traveled in CPP apparatus during priming test. Two-way ANOVA with Sidak's multiple comparisons test. M-S-Vhl group, n = 6 mice; M-S-CNO group, n = 6 mice; M-M-Vhl group, n = 7 mice; M-M-CNO group, n = 8 mice. F _(1, 23)_ = 3.759, *p* = 0.0649; M-S-CNO group, t = 0.9591, *p* = 0.9228 vs M-S-Vhl group; M-M-CNO group, t = 1.834, *p* = 0.3919 vs M-M-Vhl group; M-M-Vhl group, t = 1.623, *p* = 0.5302 vs M-S-Vhl group. Vhl, vehicle; CNO, clozapine-N-oxide; S-S, saline challenge-primed reinstatement test following saline CPP extinction training; S-M, METH challenge-primed reinstatement test following saline CPP extinction training; M-S, saline-primed reinstatement test following METH CPP extinction training; M-M, METH-primed reinstatement test following METH CPP extinction training; N.S., *p* > 0.05, *, *p* < 0.05, **, *p* < 0.01 vs S-S or M-S or Vhl or CNO.

Similarly, we conducted inhibition of eLPB^ChAT^ terminals in the ovBNST^PKCδ^ in METH-exposed mice (Figures 7A, 7B and 7C). Consistent with the findings of eLPB^ChAT^-lCeA^PKCδ^, inhibiting the eLPB^ChAT^-ovBNST^PKCδ^ pathway also had no influence on METH-primed reinstatement of CPP in mice (Figure 7D). Subsequently, we locally activated eLPB^ChAT^ terminals in the ovBNST^PKCδ^ in METH-exposed mice (Figures 7E and 7F), and found that activating eLPB^ChAT^-ovBNST^PKCδ^ pathway effectively blocked METH-primed reinstatement of METH CPP in mice (Figure 7G). We thought that the activation of eLPB^ChAT^ neurons by METH priming may represent a compensatory response of the brain, potentially attenuating sensitivity to drug priming by activating ovBNST^PKCδ^ neurons.

Taken together, these findings demonstrate that the eLPB^ChAT^-ovBNST^PKCδ^ pathway, rather than eLPB^ChAT^-CeA^PKCδ^ pathway, is encoding the METH-primed reinstatement of CPP.

## Discussion

**Figure 8:**
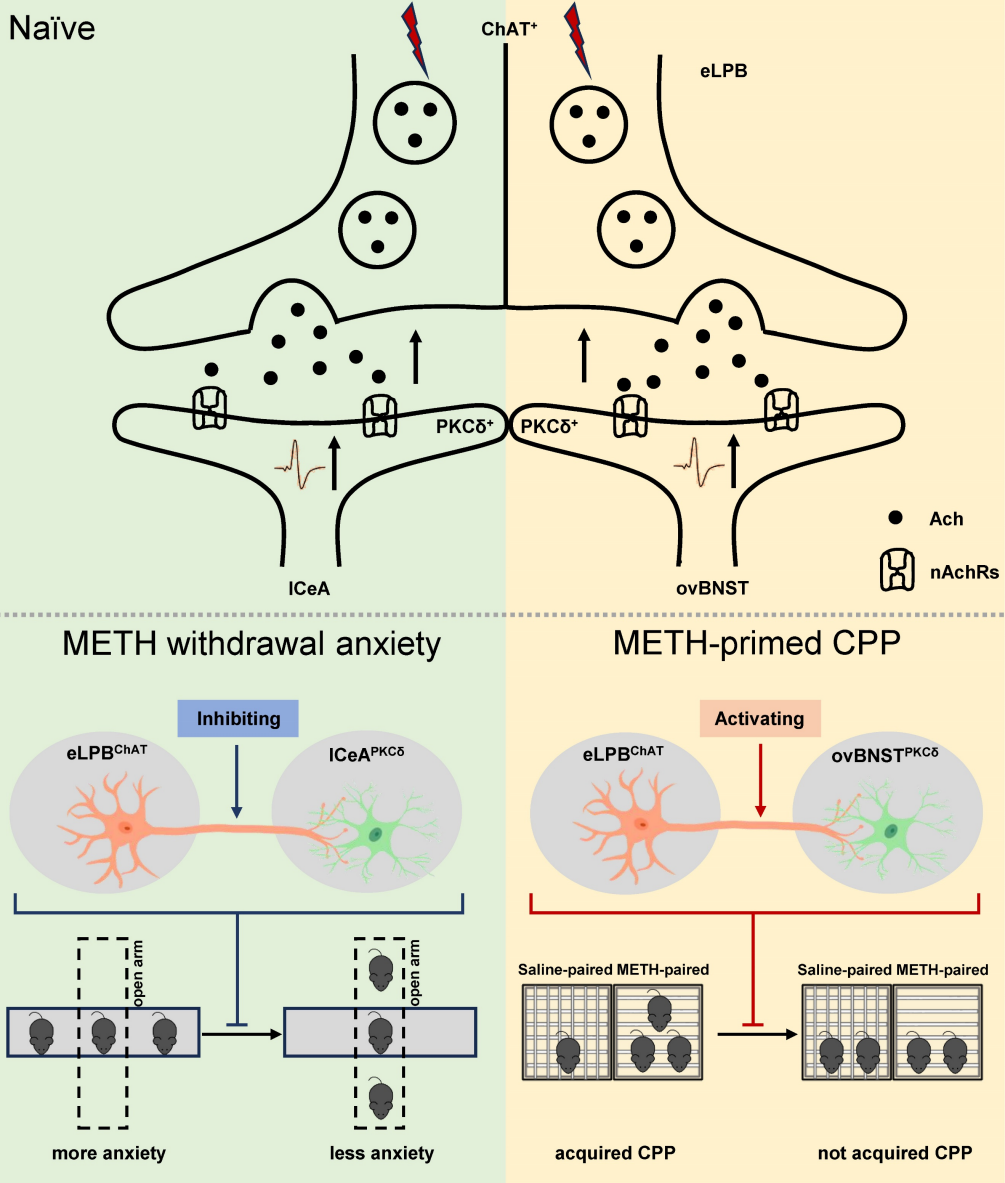
** Schematic diagram of the present study.** The ChAT^+^ neurons in the eLPB send projections to PKCδ^+^ neurons in the lCeA and ovBNST, forming eLPB^ChAT^-lCeA^PKCδ^ and eLPB^ChAT^-ovBNST^PKCδ^ pathways. At least in part, the eLPB^ChAT^ neurons positively excite the lCeA^PKCδ^ neurons and ovBNST^PKCδ^ neurons through synaptic elements of presynaptic Ach release and postsynaptic nAChRs. Chemogenetic inhibiting the eLPB^ChAT^ terminals within the lCeA alleviates the anxiety-like behaviors in METH-withdrawn mice, and chemogenetic activating the eLPB^ChAT^ terminals within the ovBNST blocks METH-primed reinstatement of CPP in METH-exposed mice. These results indicate that METH withdrawal anxiety and METH-primed relapse recruit distinct eLPB^ChAT^ projections, as identified by eLPB^ChAT^-lCeA^PKCδ^ pathway and eLPB^ChAT^-ovBNST^PKCδ^ pathway, respectively.

In summary, our study has made a novel discovery by identifying two distinct anatomically and functionally pathways of eLPB^ChAT^ neurons, namely eLPB^ChAT^-lCeA^PKCδ^ pathway and eLPB^ChAT^-ovBNST^PKCδ^ pathway. We further elucidate the involvement of synaptic elements of presynaptic Ach release and postsynaptic nAChRs in the positive innervation of the two cholinergic pathways. Importantly, our findings demonstrate that METH withdrawal anxiety and METH-primed reinstatement of CPP respectively recruit eLPB^ChAT^-lCeA^PKCδ^ and eLPB^ChAT^-ovBNST^PKCδ^ pathway in METH-exposed male mice (Figure 8).

In our previous study 14, chemogenetic activation of either the eLPB^ChAT^ neurons or the CeA-projecting eLPB^ChAT^ neurons significantly suppressed METH primed-reinstatement of CPP in mice. Here, our chemogenetic experiments specifically targeted the eLPB^ChAT^ projection to the lCeA using DREADD virus in the eLPB, followed by a site-specific infusion of the CNO into the lCeA to evaluate the effects of eLPB^ChAT^-lCeA pathway in METH primed-reinstatement. We found that chemogenetic modulation of local presynaptic activity at eLPB^ChAT^ axon terminals within the eLPB^ChAT^-lCeA pathway had no effect on METH primed-reinstatement of CPP. These seemingly contradictory findings raise significant concerns regarding whether eLPB^ChAT^ neurons projecting to other brain regions encode METH primed-reinstatement of CPP.

In addition to fMOST results 14, anterograde and retrograde tracing results in the present study also revealed an additional region of substantial innervation, namely ovBNST, which is in line with previous immunohistochemical studies 13, 31. Convincing empirical support has substantiated the pivotal role of the BNST in facilitating stress-induced reinstatement of drug seeking 32-35. For example, mimic α2a-adrenergic receptor 33 or activating pituitary adenylate cyclase-activating peptide (PACAP) systems 32 in the BNST was sufficient to induce reinstatement of cocaine. Notably, injections of the corticotropin releasing factor (CRF) receptor antagonist into the BNST attenuated stress-induced reinstatement of cocaine 36 and morphine 37 seeking, whereas no such effect was observed in the amygdala. However, the role of BNST and CeA in reinstatement of drug seeking is controversial, as conflicting findings have been reported in various studies. For example, injections of the CRF receptor antagonist into the amygdala, but not the BNST, attenuated morphine primed-reinstatement of CPP 37. Furthermore, the blockade of noradrenergic receptors in both the BNST and the CeA effectively prevented stress-induced reinstatement of cocaine seeking, but did not influence cocaine primed-reinstatement 38. Reversible tetrodotoxin (TTX) lesions of either the BNST or the CeA blocked stress-induced reinstatement of heroin seeking 35. To data, the investigation of BNST and CeA, particularly those neurons receiving eLPB^ChAT^ projections, has been sparsely documented in METH seeking. Here, our study presents the first evidence of the eLPB^ChAT^-lCeA^PKCδ^ pathway and eLPB^ChAT^-ovBNST^PKCδ^ pathway. Results showed that activating eLPB^ChAT^-ovBNST^PKCδ^ pathway, but not the eLPB^ChAT^-lCeA^PKCδ^ pathway, suppressed METH-primed reinstatement of CPP in male METH-exposed mice. In light of these findings, we wondered whether distinct outputs from the eLPB^ChAT^ mediate different components of the METH-related behavior.

Studies indicated the CeA as a target for anxiolytic agents 39, 40. The lCeA is often referred to as the “nociceptive amygdala” due to its high concentration of neurons that respond to noxious stimuli which receive nociceptive-specific information through the PBN 41, 42. The lCeA predominantly consists of PKCδ^+^ neurons 43, 44, and optogenetic activation of lCeA^PKCδ^ was anxiolytic 45. Additionally, LPB neurons projected to the lCeA, forming a functionally significant circuit involved in appetite suppression 31 and the formation of aversive memories 6. A recent study demonstrated that chemogenetic stimulation of LPB-CeA pathway heightened anxiety-like behavior 13. In this study, we found that inhibiting eLPB^ChAT^-lCeA^PKCδ^ pathway alleviated anxiety-like behaviors in METH-withdrawn male mice, indicating the eLPB^ChAT^ may serve as the primary source of lCeA^PKCδ^ relevant to METH withdrawal-induced anxiety-like behavior.

In the current study, we found that both eLPB^ChAT^ and ovBNST^PKCδ^ neurons were activated following METH-primed reinstatement of CPP, while it was to activating rather than suppressing eLPB^ChAT^-ovBNST pathway blocked METH-primed reinstatement of CPP in mice. We predict that the activation of this pathway may be a compensatory effect for the METH-primed reinstatement of METH CPP. Notably, eLPB^ChAT^ neurons were activated as early as METH withdrawal period, while ovBNST^PKCδ^ neurons were activated after METH priming rather than the METH withdrawal period. Further, we found that activating eLPB^ChAT^ neurons enhanced the Ach release within the ovBNST, as well as triggered ovBNST^PKCδ^ neurons in mice. As such, we suspect that the activation of eLPB^ChAT^ neurons is a direct result of the concomitant effect with METH exposure, while the activation of ovBNST^PKCδ^ neurons might be secondary occurrence only after METH priming during the reinstatement of METH CPP. The BNST mainly consists of GABAergic neurons, which send inhibitory projections to some addition-related brain regions, such as ventral tegmental area 46 and nucleus accumbens 47. Therefore, activating eLPB^ChAT^-ovBNST^PKCδ^ pathway has potential to enhance the inhibitory outputs from ovBNST, which might inhibit these addiction-related regions and subsequently suppress the METH-primed reinstatement of CPP.

There are several limitations in the current study. First, there is lack of female mice models to explore the potential sex differences in the impact of eLPB^ChAT^ projections on METH toxicity. Second, there is the absence of PKCδ-promoter-tagged viral tools or animal models, which hindered the precise manipulation of CeA^PKCδ^ or BNST^PKCδ^ neurons *in vivo*.

## Conclusions

Collectively, our data show that the eLPB^ChAT^ is a critical node in the neural networks governing METH withdrawal anxiety and METH primed-reinstatement of CPP through its projections to the lCeA^PKCδ^ and ovBNST^PKCδ^, respectively.

## Supplementary Material

Supplementary figures.

## Abbreviations

Ach: acetylcholine
AUC: area under the curve
CeA: central nucleus of the amygdala
BNST: bed nucleus of the stria terminalis
ChAT^+^: choline acetyltransferase
CNO: clozapine-N-oxide
CPP: conditioned place preference
CRF: corticotropin releasing factor
DREADDs: Designer receptors exclusively activated by designer drugs
eLPB: external lateral portion of parabrachial nucleus
EPM: elevated plus maze
IACUC: Institutional Animal Care and Use Committee
lCeA: lateral portion of central nucleus of amygdala
MEC: Mecamylamine
METH: methamphetamine
MUD: METH use disorders
nAChRs: nicotinic acetylcholine receptors
ovBNST: oval portion of bed nucleus of the stria terminalis
PACAP: pituitary adenylate cyclase-activating peptide
PBN: parabrachial nucleus
PFA: paraformaldehyde
PKCδ: protein kinase C delta
sEPSC: spontaneous excitatory postsynaptic currents
TTX: tetrodotoxin
WT: wild type
