# Supplementary material for: Distinct eLPBChAT projections for methamphetamine withdrawal anxiety and primed reinstatement of conditioned place preference
Source: Theranostics. 2024 Apr 29;14(7):2881–96. doi: 10.7150/thno.95383 (PMC11103501; doi:10.7150/thno.95383)
Supplement: Supplementary file 1 — Supplementary figures. [file thnov14p2881s1.pdf]

Supplementary figures and figures legends

Figure S1. Viral validation of functional innervation experiment.

Figure S2. METH-primed reinstatement of CPP in male mice.

Figure S1

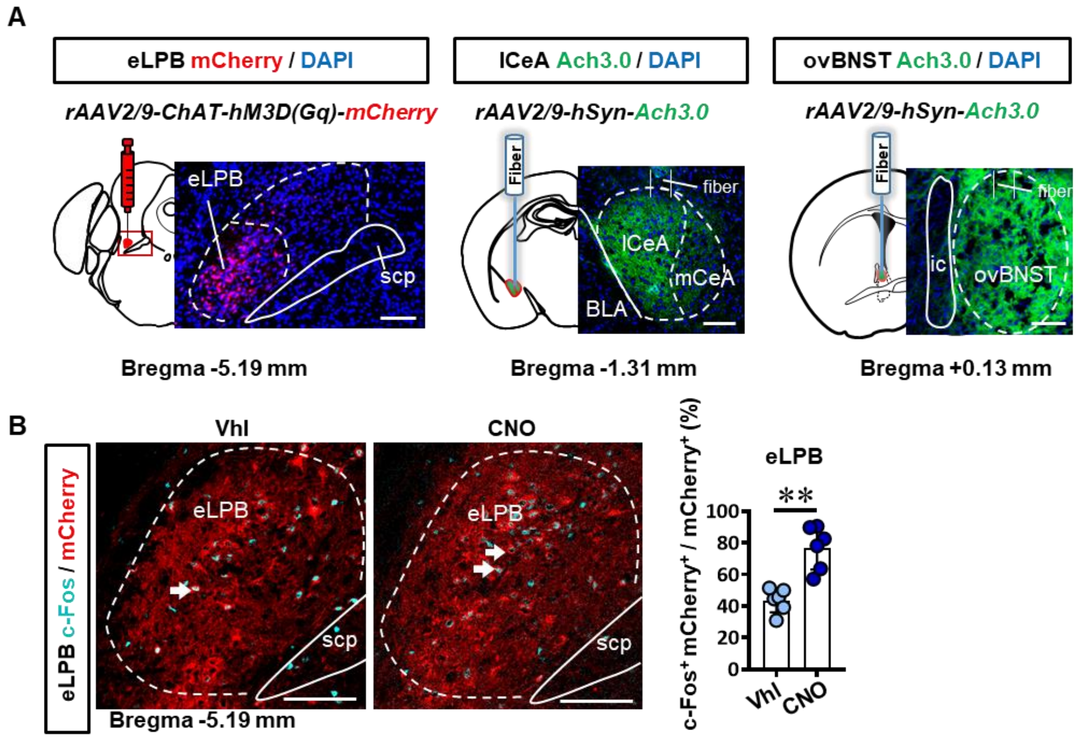
A
eLPB mCherry / DAPI
lCeA Ach3.0 / DAPI
ovBNST Ach3.0 / DAPI
rAAV2/9-ChAT-hM3D(Gq)-mCherry
rAAV2/9-hSyn-Ach3.0
rAAV2/9-hSyn-Ach3.0
eLPB
scp
Fiber
fiber
lCeA
mCeA
BLA
ic
ovBNST
Bregma -5.19 mm
Bregma -1.31 mm
Bregma +0.13 mm
B
eLPB c-Fos / mCherry
Vhl
CNO
eLPB
scp
Bregma -5.19 mm
eLPB
c-Fos+ mCherry+ / mCherry+ (%)
**
100
80
60
40
20
0
Vhl
CNO

Figure S1. Viral validation of functional innervation experiment. A Representative image of *rAAV2/9-ChAT-hM3D(Gq)-mCherry* injection sites in the eLPB and *rAAV2/9-hSyn-Ach3.0* injection sites in the lCeA and ovBNST of WT mouse. Scale bar, 100 μm. **B** Representative images and the percentage of c-Fos-positive neurons on mCherry-transfected neurons in the eLPB. Two-tailed unpaired t test. n = 6 mice per group. $t_{(10)} = 5.190$, $p = 0.0004$. ** $p < 0.01$ vs Vhl. Scale bar, 100 μm.

Figure S2

A

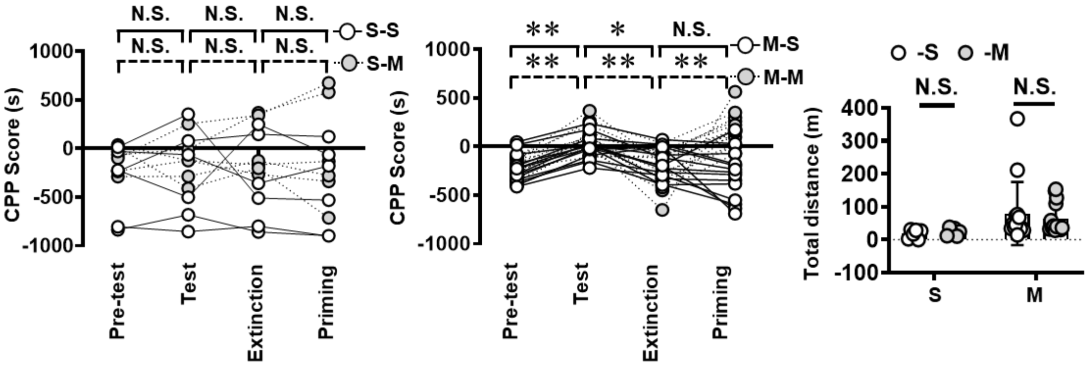
N.S.
N.S.
N.S.
N.S.
N.S.
N.S.
S-S
S-M
CPP Score (s)
1000
500
0
-500
-1000
Pre-test
Test
Extinction
Priming
**
*
N.S.
**
**
**
M-S
M-M
CPP Score (s)
-S
-M
N.S.
N.S.
Total distance (m)
400
300
200
100
0
-100
S
M

Figure S2. METH-primed reinstatement of CPP in male mice. A CPP scores during the pre-test, CPP test, extinction and priming in S-S and S-M group male mice (left) or in M-S and M-M group male mice (middle). Two-way ANOVA with Sidak's multiple comparisons test. S group, n = 6 mice per group. $F_{(3, 40)} = 0.1837$, $p = 0.9069$; S-S group priming, t = 0.2338, $p > 0.9999$ vs extinction; S-M group priming, t = 0.1149, $p > 0.9999$ vs extinction. M group, n = 14 mice per group. $F_{(3, 104)} = 14.62$, $p < 0.0001$; M-S group priming, t = 2.093, $p = 0.2111$ vs extinction; M-M group priming, t = 6.453, $p < 0.0001$ vs extinction. Total distance traveled in CPP apparatus during priming test (right). Two-way ANOVA with Sidak's multiple comparisons test. S group, n = 6 mice per group; M group, n = 14 mice per group. $F_{(1, 36)} = 0.2026$, $p = 0.6554$; S-M group, t = 0.1443, $p > 0.9999$ vs S-S group; M-M group, t = 0.6013, $p = 0.9919$ vs M-S group.

S-S, saline-primed reinstatement test following saline CPP extinction training; S-M, METH-primed reinstatement test following saline CPP extinction training; M-S, saline-primed reinstatement test following METH CPP extinction training; M-M, METH-primed reinstatement test following METH CPP extinction training; N.S., $p > 0.05$, *, $p < 0.05$, **, $p < 0.01$.
